# Physiological Epidermal Growth Factor Concentrations Activate High Affinity Receptors to Elicit Calcium Oscillations

**DOI:** 10.1371/journal.pone.0106803

**Published:** 2014-09-29

**Authors:** Béatrice Marquèze-Pouey, Sébastien Mailfert, Vincent Rouger, Jean-Marc Goaillard, Didier Marguet

**Affiliations:** 1 Centre d’Immunologie de Marseille-Luminy, UM2 Aix Marseille Université, Marseille, France; 2 INSERM, U1104, Marseille, France; 3 CNRS, UMR7280, Marseille, France; 4 INSERM, UMR_S 1072, Marseille, France; 5 Aix-Marseille Université, UNIS, Marseille, France; China Agricultural University, China

## Abstract

Signaling mediated by the epidermal growth factor (EGF) is crucial in tissue development, homeostasis and tumorigenesis. EGF is mitogenic at picomolar concentrations and is known to bind its receptor on high affinity binding sites depending of the oligomerization state of the receptor (monomer or dimer). In spite of these observations, the cellular response induced by EGF has been mainly characterized for nanomolar concentrations of the growth factor, and a clear definition of the cellular response to circulating (picomolar) concentrations is still lacking. We investigated Ca^2+^ signaling, an early event in EGF responses, in response to picomolar doses in COS-7 cells where the monomer/dimer equilibrium is unaltered by the synthesis of exogenous EGFR. Using the fluo5F Ca^2+^ indicator, we found that picomolar concentrations of EGF induced in 50% of the cells a robust oscillatory Ca^2+^ signal quantitatively similar to the Ca^2+^ signal induced by nanomolar concentrations. However, responses to nanomolar and picomolar concentrations differed in their underlying mechanisms as the picomolar EGF response involved essentially plasma membrane Ca^2+^ channels that are not activated by internal Ca^2+^ store depletion, while the nanomolar EGF response involved internal Ca^2+^ release. Moreover, while the picomolar EGF response was modulated by charybdotoxin-sensitive K^+^ channels, the nanomolar response was insensitive to the blockade of these ion channels.

## Introduction

EGF controls key cellular processes, such as proliferation, survival, differentiation during development, tissue homeostasis, and tumorigenesis (reviewed in [1]). Through binding to the tyrosine kinase EGF receptor (EGFR), EGF activates a wide variety of signaling cascades mostly leading to the regulation of gene transcription. EGF is synthesized as a transmembrane precursor from which a mature, diffusible form is generated by metalloproteases. Soluble EGF can activate EGFR on distant cells via an endocrine/paracrine pathway or cells of its origin via an autocrine action. Endocrine/paracrine EGF is mitogenic at picomolar concentrations. Human serum contains around 40 pM EGF [2], which is active on cell proliferation at a twenty-fold dilution [3].

Consistent with these findings, high affinity classes of EGF binding sites have been demonstrated to be present at the cell surface. Analysis of ^125^I-EGF binding data [4] combined with crystallographic structures of drosophila [5] and human [6], [7] EGFRs have suggested that the proposed high-affinity and low-affinity classes of EGF binding sites at the cell surface reflect negative cooperative binding to dimeric forms of the receptor, high affinity sites being the unliganded dimer, and low affinity sites the dimer already bound to one molecule of EGF. Recent data [8] also suggested that monomers carry low affinity binding sites so that the high affinity sites could be a dimeric receptor already preformed and primed for fast activation by EGF.

Most studies on EGFR signaling have focused on low affinity binding site receptors as EGF concentrations used were in the nanomolar range. However, such doses might only be reached in autocrine signaling in the immediate vicinity of cell-surface receptors or in juxtacrine activation with a non-diffusible transmembrane ligand engaged with EGFR on adjacent cell membrane. Endocrine and paracrine responses to EGF are likely to mainly involve binding to high affinity EGFR sites. So we asked whether EGF at plasmatic concentrations, compatible with the binding to EGFR high affinity binding sites, was able to induce a significant cellular response. We chose to analyze Ca^2+^ signaling, an early event in EGF responses already thoroughly characterized for nanomolar doses [9]. We used COS-7 cells naturally expressing endogenous EGF receptors [10], in order to ensure that the monomer/dimer equilibrium was unaltered, in contrast to A431 carcinoma cells [9], [11] or EGFR-transfected fibroblasts [10].

Using sensitive microscope-based real time imaging of calcium dynamics, we unexpectedly discovered that plasmatic concentrations of EGF (less than 20 pM) induce a distinctive robust oscillatory Ca^2+^ signaling mode quantitatively comparable to the Ca^2+^ signal obtained in response to nanomolar concentrations. However, the two responses were qualitatively different as picomolar EGF response involved essentially plasma membrane Ca^2+^ channels that are not activated by store depletion, but was modulated by charybdotoxin-sensitive K^+^ channels. In contrast, the response to nanomolar concentrations involved internal Ca^2+^ release and was insensitive to K^+^ channel blockade.

## Material and Methods

### Cell culture

COS-7 cells (American Type Culture Collection) derived from the kidney of the African Green Monkey, *Cercopithecus aethiops* were cultured in Dulbecco's modified Eagle's medium (DMEM; Life Technologies) containing 10% (V/V) FBS (Sigma-Aldrich) at 37°C under an atmosphere of 5% CO_2_ and plated at a density of about 1.3 10^4^ cells/cm^2^ on LabTek #1 borosilicate chambered slides (Thermo Fisher Scientific) previously coated with fibronectin (BD Biosciences) at a density of 1µg/cm^2^ for 1 hour and washed with Ca^2+^-free HBSS (Life Technologies). Cells were grown for 48–72 hours and deprived of serum 18 hours before imaging in DMEM-f12 medium without phenol red (Life Technologies). We ensured that cells were healthy by culturing low-passage cell lines, by systematically evaluating cell proliferation rate from growth curve data and by verifying cellular morphology before EGF stimulation experiments.

### Drugs

Mouse EGF (Life Technologies) was diluted in fresh imaging buffer solution (3 mM Ca^2+^ in the extracellular medium, 3 mM Ca^2+^_o_); 125 mM NaCl, 2.5 mM KCl, 1.1 mM NaH_2_PO_4_, 4 mM NaHCO_3_, 2 mM MgCl_2_, 3 mM CaCl_2,_ 10 mM glucose, 10 mM Hepes, 0.2% (W/V) BSA or in Ca^2+^ free buffer (nominally 0 mM Ca^2+^ in the extracellular medium, 0 mM Ca^2+^_o_) solution; 125 mM NaCl, 2.5 mM KCl, 1.1 mM NaH_2_PO_4_, 4 mM NaHCO_3_, 2 mM MgCl_2_, 10 mM glucose, 10 mM Hepes, 0.2% (W/V) BSA, 1 mM EGTA (Sigma). EGF solutions of 40 pM and 4 nM (to be applied volume to volume to yield final concentrations of 20 pM and 2 nM, respectively) were kept on ice at all times and warmed up to 30°C just before stimulation to avoid degradation of EGF.

EGFR-specific neutralizing monoclonal M225 antibodies [12] were chosen for their capacity to inhibit EGF binding, EGFR tyrosine kinase and proliferative activities and their use in cancer therapy (humanized version Cetuximab/C225). 40 µl of 100 µg/ml solutions of anti EGFR Ab-3 mouse M225 (Calbiochem, Merck Millipore) or IgG1 were added to 400 µl cell culture chamber 200 s after the start of time-lapse recording.

Charybdotoxin (Alomone labs), a blocker of Ca^2+^-activated K^+^ channels K_Ca1.1_ [13], K_Ca3.1_ [14] and voltage-dependent K_v1.3_ channels [15] was added to cells at a concentration of 100 nM, 20 min before starting the time-lapse recording.

### Intracellular Ca^2+^ imaging

Fluo5F-AM (Life Technologies) was chosen as Ca^2+^ indicator in view of the following considerations: ***i*** *)* high quantum efficiency upon binding Ca^2+^ (comparable to Fluo4 quantum efficiency, φ  = 0.14, [16]), ***ii*** *)* excitation by light in the blue spectral range, which reduces cell phototoxicity, ***iii*** *)* low affinity for Ca^2+^ (K_D_:700 nM at 30°C, 1 mM Mg^2+^ [17]), which is important to avoid dye saturation, exogenous buffer effects [18], and to obtain an accurate estimate of the timecourse of Ca^2+^ decay.

Cells were loaded with 3.5 µM fluo5F-AM in 0.5PBX Signal Enhancer (Becton Dickinson Biosciences) and 0.5 DMEM-f12 medium without phenol red for 20 min at 37°C and 20 min at room temperature. Loaded cells were washed and equilibrated with fresh imaging buffer solution at 30°C. Video time-lapse was started and 25 s after, EGF was applied volume to volume, leading to final concentrations of 20 pM and 2 nM.

Digital fluorescence images were obtained using an inverted Axio Observer.Z1 Zeiss microscope (40× oil-immersion lens; numerical aperture, 1.4) equipped with an image acquisition system (Axiovision, Zeiss), a metal halide HXP-120 lamp for fluorescence excitation and a GFP fluorescence cube (Zeiss, filter set 38 HE). To reduce photodamage, neutral density filters were introduced in the light path to illuminate samples with 380 µW as measured at the back aperture of the objective. Images were acquired with a CCD camera, (Photometrics HQ2, interline transfer chip (1392×1040 pixels) reaching a final pixel size of 322 nm after ×40 magnification and a 2× binning to obtain a good signal to noise ratio with minimal illumination of the cells. Temperature was maintained at 30°C to slow down loss of cytoplasmic Ca^2+^ indicator [19]. The standard protocol to study Ca^2+^ transients consisted in acquiring a sequence of 300 images with an integration time of 68 ms, and a 1s interval between consecutive images.

### Image analysis

**Figure 1:**
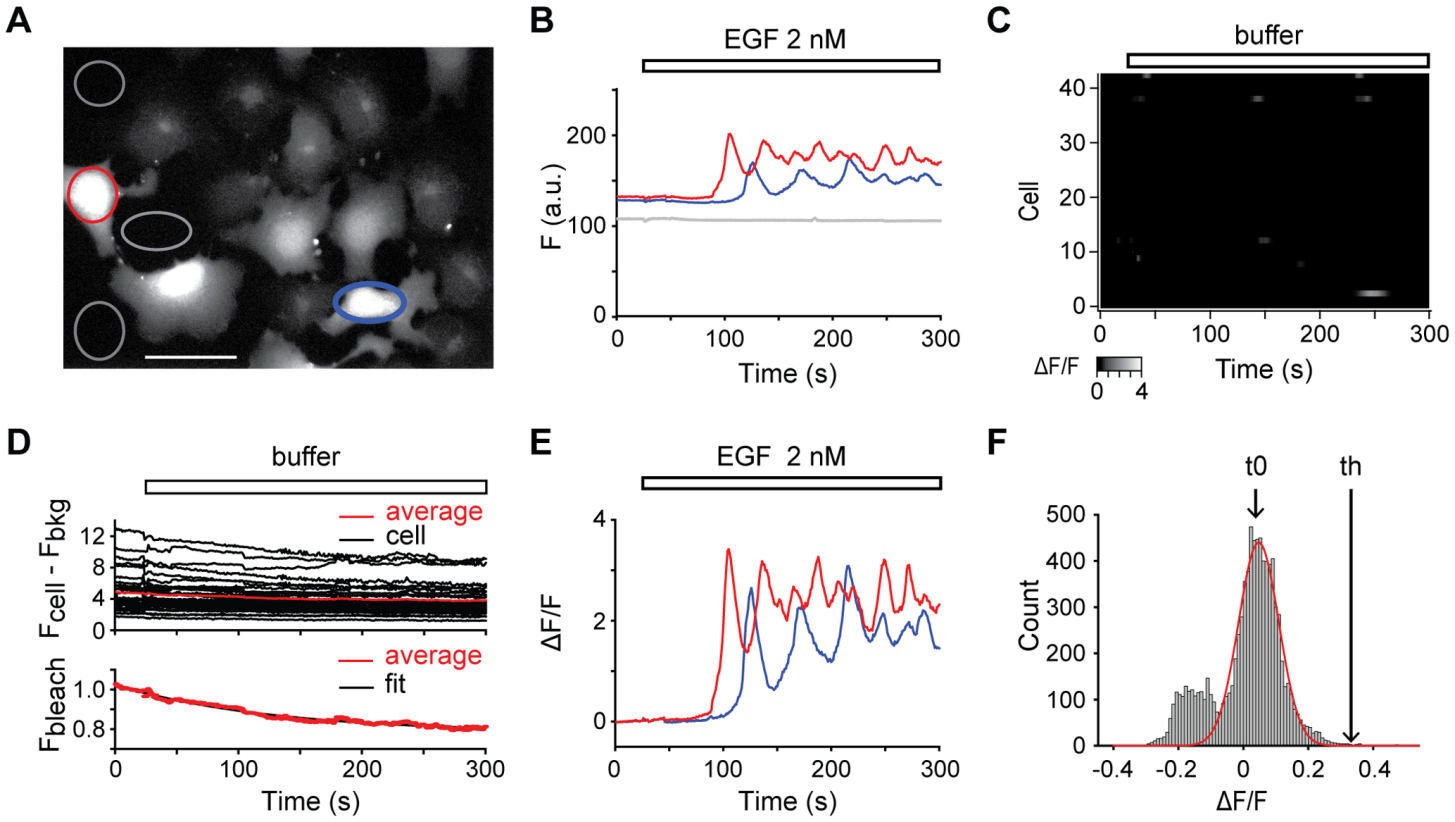
Image analysis protocol to study Ca^2+^ signaling. A/Image of fluo5F calcium-dependent fluorescence after addition of 2 nM EGF. ROIs were drawn over two COS-7 cells (red and blue circles) and 3 areas outside cells (grey circles) in the same visual field. Scale bar: 100 µm. B/Raw fluorescence intensity (*F*) as a function of time for the two cell ROIs (red and blue lines), and the three background ROIs (grey lines) shown in A. 2 nM EGF application (represented by a white bar) was performed 25 s after the start of the video time-lapse. C/Grayscale-coded raster plot of fluorescence intensity over time of 41 control cells in response to buffer application (*i.e.* in the absence of EGF). Buffer was added 25 s after the start of the video time-lapse (as indicated by a white bar). D/Data (upper graph) obtained in cells subjected to control application of buffer in the absence of EGF (same data as in C). Background fluorescence (*F_bkg_*), evaluated by averaging the fluorescence of three areas outside cells, was subtracted from the signal (*F_cell_*), measured in 32 cells showing no fluorescence peak throughout the entire video time-lapse. The average fluorescence did not exhibit a flat baseline due to photobleaching. An *F_bleach_* term was determined from a single exponential fit (lower graph) to the average of 32 traces calculated from *F_cell_* -*F_bkg_*/*F*(0) where *F*(0) is the average of the 25 images preceding buffer application (white bar, 25 s after the start of the video time-lapse). E/Normalized fluorescence intensity (Δ*F*/*F*) as a function of time for the two cell ROIs (red and blue lines) shown in A. EGF (white bar) was added to cells 25 s after the start of the video time-lapse. F/Histogram of fluorescence intensity values from the 32 control cells where no peak was detected when buffer was added. A centered value (*t*0) and a standard deviation (*SD*) were extracted from the Gaussian fit (red line) of the distribution and a threshold value (*th*) was set as *t*0+3 *SD* = 0.23, and was used for the detection of significant responses in further experiments.

Fig 1A illustrates the pattern of fluorescence reported by fluo5F in COS-7 cells after addition of 2 nM EGF. Fluo-5F emission (max 516 nm) changes were analyzed off-line with the Image J software to measure the average fluorescence of individual cells (*F_cell_*) within a “region of interest” (ROI) covering the cell area (Fig 1A). The mean intensity over the same area for the successive images of the stack was then measured automatically to monitor the timecourse of fluorescence changes. Approximately 10 to 20 cells were measured in each field of view. Background fluorescence was evaluated by averaging fluorescence (*F_bkg_*) in three regions of similar size to the cellular ROIs, which were located in the periphery of the recorded field (Fig 1A and B), and was subtracted from the fluorescence value.

The changes in fluorescence (Δ*F*(*t*) = *F*(*t*) – *F*(0)) were calculated (Sigma Plot software) where *F*(*t*) is the fluorescence at any time and *F*(0) the average of the 25 images preceding the addition of EGF. To correct for non-uniformities in dye concentration [18], Δ*F*(*t*) was divided by the pre-stimulus fluorescence. Thus, the ratio was: Δ*F*(*t*)/*F*(0) = (*F*(*t*) – *F*(0))/*F*(0).

Control experiments were performed for time-dependent dye bleaching over the course of the experiment. In 41 control cells, EGF-free buffer was added (Fig. 1C). Fluorescence was monitored in 32 cells showing no fluorescence peak throughout the entire time-lapse recording (Fig 1D) and was found to exhibit a decaying baseline. Therefore, an *F_bleach_* term was determined by fitting the average of the fluorescence signal in these 32 cells ((*F_cell_* – *F_bkg_*)/*F*(0)) with a single exponential function (*F* = 0.78+0.24*e* ^(−0.008*t*)^, time constant = 130s, Fig. 1D). The exponential fit was then used to correct the fluorescence signal in responsive cells for dye bleaching: *F*(*t*) values were normalized with the exponential function *F*(*t*)*_bleach_* to produce the Δ*F*/*F* traces (Fig. 1E). This ratio is a unique function of the stimulus-induced change in intracellular Ca^2+^ (Ca^2+^_i_).

**Figure 2:**
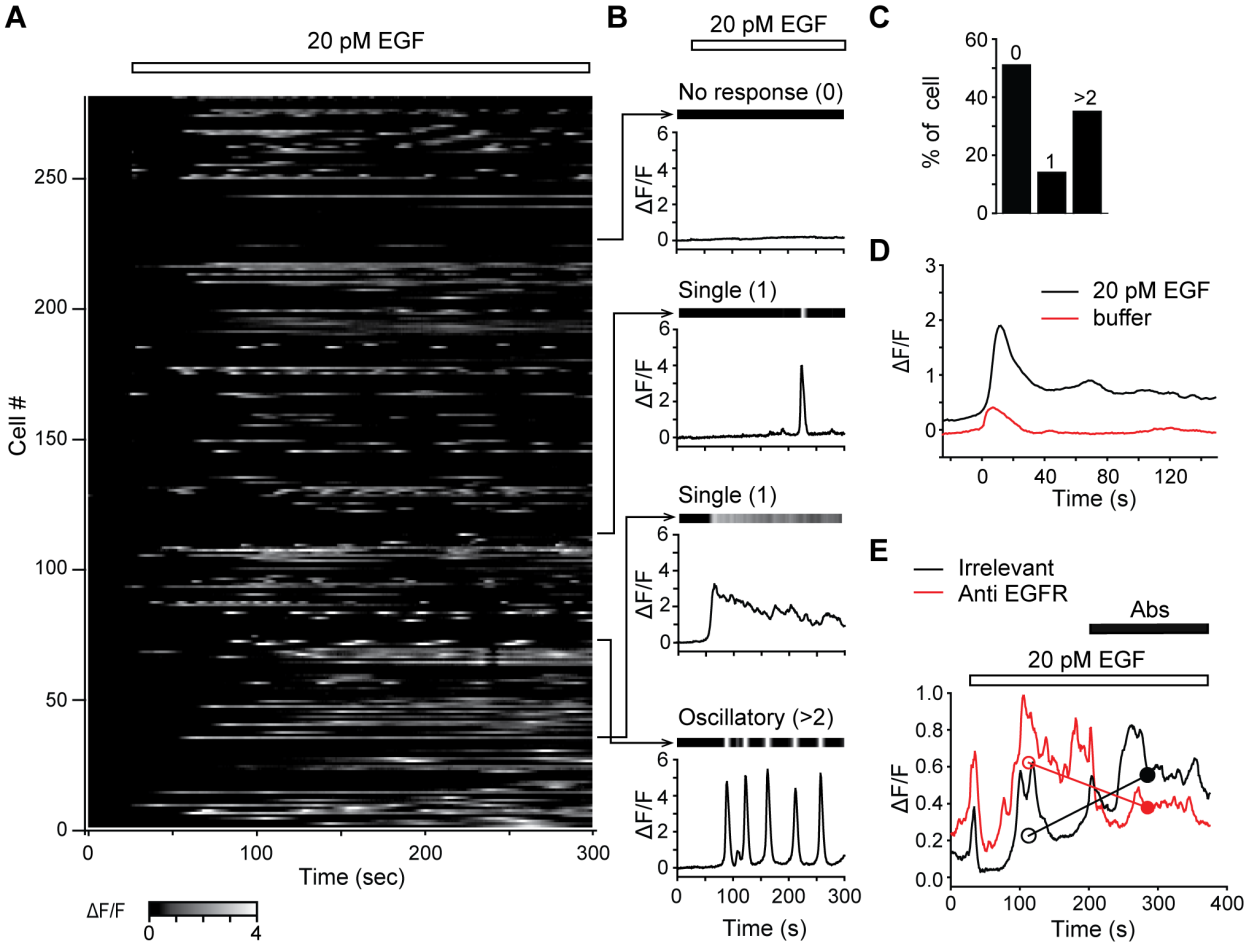
Ca^2+^ single-cell microscopy measurements induced by 20 pM EGF in COS-7 cells. A/Raster plot of normalized fluorescence intensity against time, grayscale coded according to fluorescence intensity. 20 pM EGF was applied 25 s after the start of the video time-lapse (white bar). B/Representative traces of fluorescence variation over time for four individual cells corresponding to the three classes of responses observed following 20 pM EGF application: from top to bottom panels, unresponsive cell (0 peak); cells displaying transient or sustained single response (1 peak); cell displaying oscillatory signals (>2 peaks). For each cell, the response is represented both as a grayscale coded raster plot (top, same representation as in A) and as line plot (bottom). C/Proportion of unresponsive (0 peak), single-peak responsive (1 peak) and oscillatory responsive (>2 peaks) cells following the addition of 20 pM EGF. D/Comparison of the average fluorescence signals in response to the addition of EGF-free buffer (n = 8 responsive cells over 41 tested, red trace) or of 20 pM EGF (n = 137 over 281 tested, black trace). Fluorescence signals were synchronized at the time the first fluorescence slope (time = 0 s), found by estimating the first derivative of the signal, and averaged over 150 s. E/Ca^2+^ signals are specifically triggered by EGFR activation. Population traces averaged over cells to which irrelevant (n = 32, black line) or antagonistic anti-EGFR (n = 19 cells, red line) antibodies were added (black bar) 200 s after the start of real-time fluorescence imaging. Empty and filled circles represent the median intensity during 176 s before and after the addition of antibodies respectively. EGF was applied 25 s after the start of the video time-lapse (white bar).

Cell responsiveness to EGF was determined by selecting cells that displayed calcium signals rising above an amplitude threshold (*th*) which was defined from the distribution of normalized fluorescence intensity values from the 32 non-responsive control cells subjected to buffer in the absence of EGF: a mean (*t*0) of 0.047 and a standard deviation (*SD*) of 0.06 were extracted from a Gaussian fit curve of the values and *th* was set as *t*0+3*SD* (Fig. 1F). The calculated *th* value was 0.23, high enough to exclude the appearance of false positives. With these detection criteria, 93% (40/43) cells responded to 2 nM EGF and 49% (137/281 cells) to 20 pM EGF. Signals described as oscillating in Fig. 2C displayed at least 2 peaks during the time frame.

**Figure 3:**
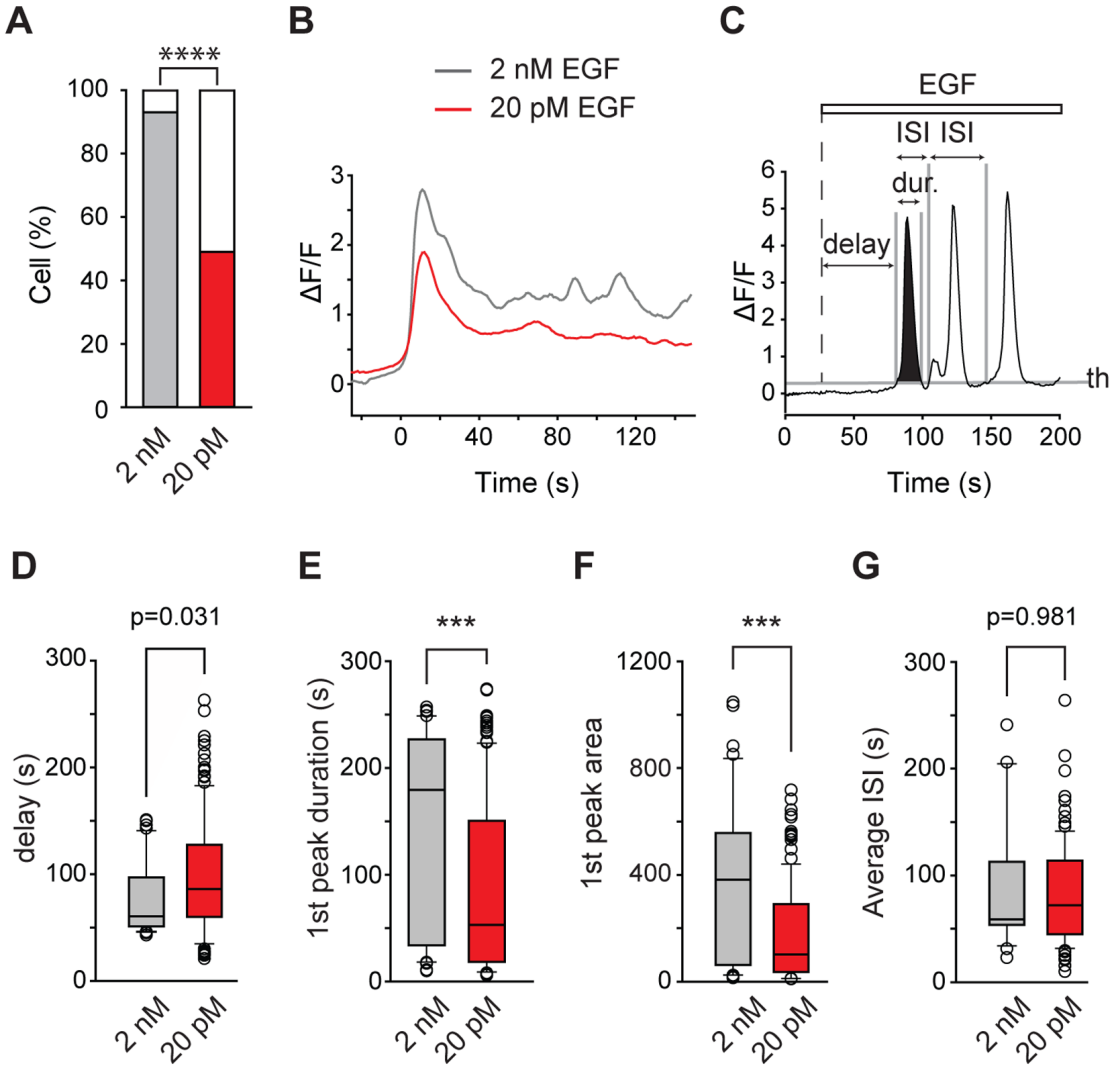
Comparative analysis of the Ca^2+^ responses following application of EGF at two different concentrations. A/Proportion of cells not responding (white bar) or responding to 2 nM (grey bar, n = 40/43) or 20 pM (red bar, n = 137/281) EGF. B/Average of responsive cell Ca^2+^ signals time-locked on the first fluorescence peak and recorded over 150 sec in response to 2 nM (grey line, n = 40) or 20 pM (red line, n = 137) EGF application. C/Schematic representation of the rules used to define the properties of the fluorescence peaks during an oscillatory response. Peaks were defined as signals rising and falling through an intensity threshold (th) of 0.23, and delay, duration and inter-spike interval (ISI, difference between the starting time of 2 consecutive peaks) values were defined relative to the threshold crossing. The area under the first peak is shown in black. EGF was added 25 s after the start of the video time-lapse (white bar). D/Bar plot showing the distribution of first peak delays as defined in C elicited by 2 nM (grey box, n = 40/43) or 20 pM (red box, n = 137/281) EGF. E/Bar plot showing the distribution of first peak durations as defined in C elicited by 2 nM (grey box, n = 40/43) or 20 pM (red box, n = 137/281) EGF. F/Bar plot showing the distribution of first peak areas as defined in C elicited by 2 nM (grey box, n = 40/43) or 20 pM (red box, n = 137/281) EGF. G/Bar plots showing the distribution of average interspike intervals (Average ISI) for oscillatory cells responding to 2 nM (grey box, n = 22/43 cells) or 20 pM (red box, n =  98/281 cells) EGF.

To quantify oscillatory properties in single cells (Fig. 3 D–G), responses rising and falling through the *th* level were detected as “spikes” using custom software developed in IGOR. “Spike” duration and area were calculated. Peaks with a duration shorter than 10 s and an area smaller than 10 (a.u.) were regarded as noise oscillations around threshold and discarded.

### Data analysis

Kinetics of signals from responsive cells (Fig 2D and 3B) were obtained by averaging all cell signals, synchronized at the time of the first rise in fluorescence, for 150 s.

**Figure 4:**
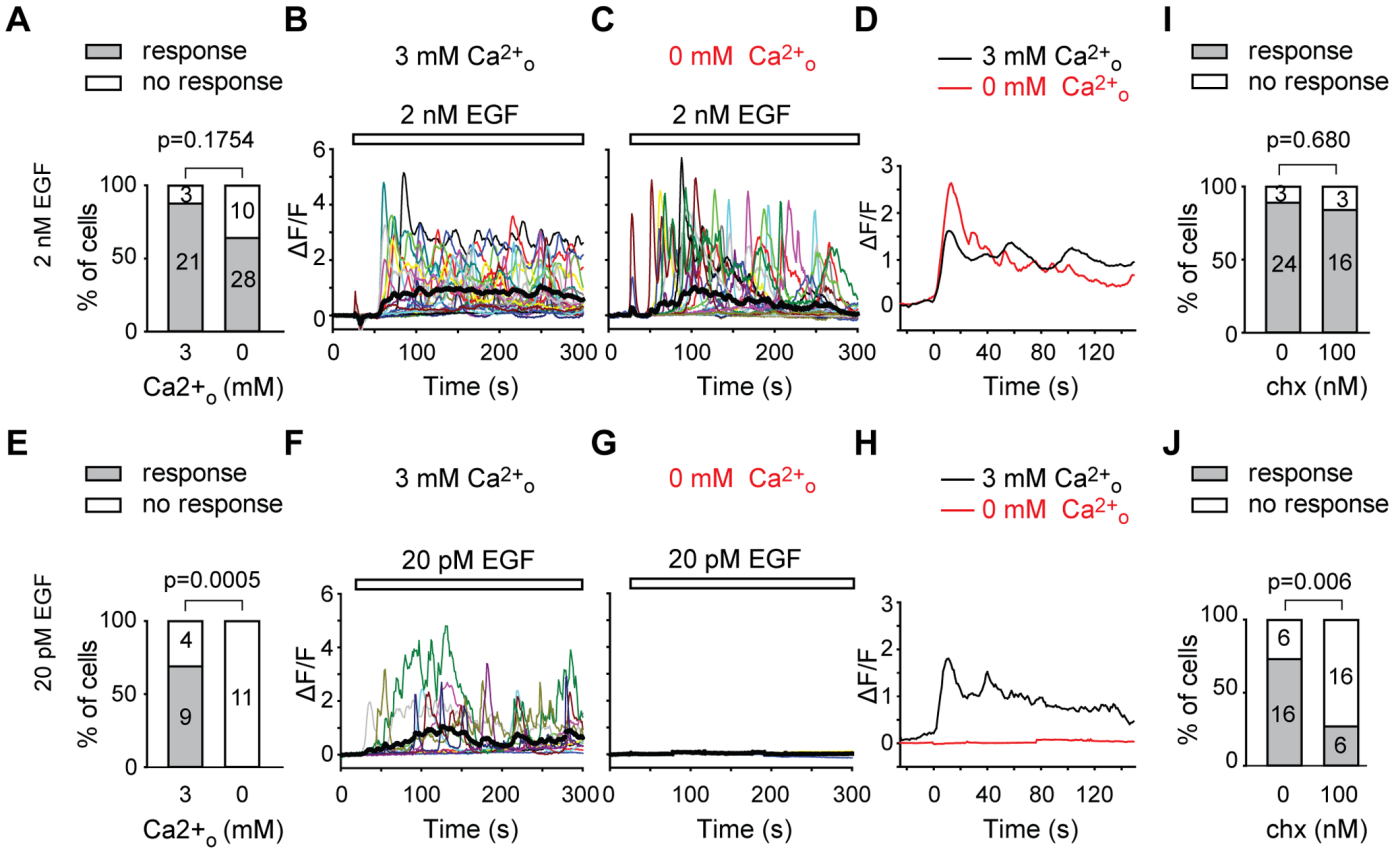
External Ca^2+^ dependence and sensitivity to K^+^ channel blocker charybdotoxin of EGF Ca^2+^ transients. A/Proportion of cells responding (grey bar) or not responding (white bar) to 2 nM EGF in 3 mM extracellular Ca^2+^ (n = 24) or in 0 mM Ca^2+/^1 mM EGTA (n = 28) in the extracellular medium. B/Fluorescence intensity signaling of individual cells (each represented by a different color) during the application of 2 nM EGF (white bar) when 3 mM Ca^2+^ was present (n = 24). The averaged population signal is shown as a thick black trace. C/Fluorescence intensity of individual cells (each represented by a different color) during the application of 2 nM EGF (white bar) when Ca^2+^ was removed and 1 mM EGTA was added to the extracellular medium (n = 28). The averaged population signal is shown as a thick black trace. D/Average of all cell signals during 2 nM EGF application, synchronized at the time of the first fluorescence peak and averaged for 150 sec, when 3 mM Ca^2+^ was present (black line, n = 24) or when Ca^2+^ was removed from and 1 mM EGTA was added to the extracellular medium (red line, n = 28). E/Proportion of cells responding (grey bar) or not responding (white bar) to 20 pM EGF in 3 mM extracellular Ca^2+^ (n = 13) or in 0 mM Ca^2+/^1 mM EGTA (n = 11) in the extracellular medium. F/Fluorescence intensity of individual cells (each represented by a different color) during the application of 20 pM EGF (white bar) when 3 mM Ca^2+^ was present (n = 13). The averaged population signal is shown as a thick black trace. G/Fluorescence intensity of individual cells (each represented by a different color) during the application of 20 pM EGF (white bar) when Ca^2+^ was removed from and 1 mM EGTA was added to the extracellular medium (n = 11). The averaged population signal is shown as a thick black trace. H/Average of all cell signals during 20 pM EGF application, synchronized at the time the first fluorescence peak and for 150 sec, when 3 mM Ca^2+^ was present (black line, n = 13) or when Ca^2+^ was removed from and 1 mM EGTA was added to the extracellular medium (red line, n = 11). I/Proportion of cells responding (grey bar) or not responding (white bar) to 2 nM EGF in the absence (0, n = 24/27) or in the presence (100, n = 16/19) of 100 nM charybdotoxin (chx) in the extracellular medium. J/Proportion of cells responding (grey bar) or not responding (white bar) to 20 pM EGF in the absence (0, n = 16/22) or in the presence (100, n = 6/22) of 100 nM charybdotoxin (chx) in the extracellular medium.

To determine the effect of extracellular Ca^2+^ on EGF responses (Figs. 4B–C and 4F–G), an average signal was generated from the entire single-cell signals over the 300 s time frame and an average curve was superimposed in bold on individual traces.

Statistical analysis was performed according to the distribution properties of the data, by rank Wilcoxon and Mann–Whitney tests, Fisher's exact test (for contingency table analysis), Gaussian fitting procedure (all conducted using SigmaPlot 11.0, Jandel Scientific), with p <0.001 considered to be statistically significant. Figures were prepared using SigmaPlot, Igor Pro, and Adobe Illustrator CS5. Unless otherwise stated, data distributions are presented as median values. Box and whisker plots are represented with the first and third quartiles at the ends of the box, the median is indicated by a horizontal line in the box, the 5^th^ and 95^th^ percentiles are marked with a bar at the ends of the whiskers and outliers are shown as open circles. Statistical significance of p<0.001 and p<0.0001 are indicated on graphs by the use of ***, and **** symbols.

## Results

### Picomolar concentrations of EGF elicit oscillatory Ca^2+^ responses

Intracellular calcium (Ca^2+^_i_) dynamics induced by EGF in COS-7 cells were studied by measuring changes in fluorescence intensity of the low affinity Ca^2+^ indicator fluo5F, which faithfully reports kinetics in single living cells [20]. Ca^2+^_i_ variations were quantified as illustrated in Fig. 1. Exposure of COS-7 cells to 2 nM EGF resulted in a rise in Ca^2+^ levels subsequently reaching a plateau (Fig. 1E), consistent with the responses to 10 nM EGF previously reported using fura-2 calcium imaging [9].

Ca^2+^ signals in response to 20 pM EGF were then characterized in 281 cells (Fig. 2). Forty nine percent of cells responded to 20 pM EGF by producing a significant Ca^2+^ signal. Although high cell-to-cell variability in the Ca^2+^_i_ timecourse was observed, the majority of responsive cells (72%) displayed an oscillatory Ca^2+^ signal (Fig. 2A– C). As these responses were triggered by an unusually low concentration of EGF, we verified that they were specific to EGF application. Non-specific calcium fluctuations were quantified by applying EGF-free buffer. Data from 41 cells (raster plot in Fig. 1C displays the intensity of fluorescence, encoded in grayscale, over time) were averaged over 150 s after the first rise in fluorescence and compared with those elicited in the presence of 20 pM EGF (Fig. 2D). To quantify the difference between the Ca^2+^ responses in the absence and in the presence of 20 pM EGF, Ca^2+^ load into the cells was defined by measuring the area under the fluorescence curve. The area in response to the addition of EGF-free buffer was found to be 0.6% of the area in response to 20 pM and considered negligible. In order to confirm that responses to 20 pM EGF were specific to EGFR activation, the effects of antagonistic anti-EGFR antibodies versus irrelevant IgG1 antibodies (isotype control) were determined. While the average fluorescence intensity values steadily increased after irrelevant antibodies were added (n = 32 cells), possibly related to an application effect that was also seen immediately after buffer (Fig. 2D) or EGF (Fig. 2E) applications, a significant decrease (Wilcoxon test, p<0.001) was seen when anti-EGFR antibodies were applied (Fig. 2E): median values of the fluorescence signal before and after anti-EGFR antibodies were respectively 0.62 and 0.38, demonstrating the specificity of the Ca^2+^ response to 20 pM EGF.

### Picomolar and nanomolar concentrations of EGF elicit comparable Ca^2+^ responses

A statistical comparison of the Ca^2+^ responses to 2 nM and 20 pM EGF was performed (Fig. 3). While a higher fraction of cells (Fig. 3A) responded to 2 nM than to 20 pM EGF (93%, n = 40/43 *vs* 49%, n = 137/281; Fisher's exact p<0.0001), no noticeable differences were found in the kinetics of the averaged Ca^2+^ signal (first peak rise and decay, Fig. 3B) in response to 2 nM or 20 pM. Considering the ratio of the concentrations applied (2 nM/20 pM = 100), the difference in the intensity of the calcium signal elicited by the two concentrations was rather modest (1.2 *vs* 0.7 for 2 nM and 20 pM, respectively, ratio = 1.7), although statistically significant (p<0.001, Mann-Whitney).

To analyze the properties of the oscillatory responses observed in response to 2 nM and 20 pM EGF, we defined a peak as a signal that rises and falls through the intensity threshold *th* (Fig. 3C), calculated from the Gaussian distribution of fluorescence intensity values in control experiments where EGF-free buffer was added to cells (Fig. 1F). Then statistical analysis was performed to determine whether the values of the different parameters characterizing the oscillatory response (delay of appearance of the first peak after EGF application, duration of the first peak, area of the first peak and the average interval between the peaks or ISI) were significantly different between the 20 pM and 2 nM EGF applications. The delays (Fig. 3D) of the responses were slightly but significantly smaller for 2 nM EGF than for 20 pM (61 *vs* 86 s, p = 0.031, Mann-Whitney). Peak duration and area were evaluated on the first peak elicited after EGF addition to avoid measurement errors on peaks exceeding the recording time frame. Peak durations (Fig. 3E) obtained at 2 nM EGF were significantly longer than those obtained at 20 pM EGF (179 *vs* 53 s, p<0.001, Mann-Whitney). Peak areas (Fig. 3F) were also significantly larger in response to 2 nM EGF compared to 20 pM EGF (382 *vs* 102, p<0.001, Mann-Whitney). However, the medians of the interspike intervals (Fig. 3G) were not significantly different at the two EGF concentrations (59 *vs* 72 s for 2 nM and 20 pM, respectively, p = 0.981, Mann-Whitney). Overall, quantifications suggest that 2 nM EGF elicit a modestly but significantly larger increase in Ca^2+^ load than 20 pM EGF, while Ca^2+^ signal kinetics are not significantly different.

### The Ca^2+^ sources involved in the EGFR response are different for picomolar and nanomolar concentrations

It is already known that Ca^2+^ signaling in response to nanomolar EGF has two components: Ca^2+^ release from the intracellular stores sequentially due to phospholipase (PLC) γ activation, inositol 1,4,5-trisphosphate (IP3) synthesis and IP3 receptor activation, and a net Ca^2+^ influx from the outer medium due to store-operated channels (SOC) [10] and/or non-SOC [21] responsible for the plateau phase. Consistent with these previous observations, when Ca^2+^ was omitted from the extracellular medium and 1 mM EGTA was added (nominally 0 mM Ca^2+^ in the extracellular medium), most cells still responded to 2 nM EGF (Fig. 4A, 88% n = 24 in 3 mM Ca^2+^_o_ vs 64% n =  28 in 0 mM Ca^2+^_o_). Also, single-cell responses appeared very similar whether Ca^2+^ was present or not in the extracellular medium. Average fluorescence curves (shown in bold lines in Fig. 4B and 4C) were comparable over the first 200 s phase, then the signal decreased in the absence of Ca^2+^_o_ while it stayed at a plateau with 3 mM Ca^2+^_o_. Furthermore, the average kinetics of the first Ca^2+^_i_ peak elicited by 2 nM EGF were very similar with or without external Ca^2+^ (Fig. 4D, compare black curve 3 mM Ca^2+^_o_ with red curve 0 mM Ca^2+^_o_). In contrast, responses to 20 pM EGF were totally abolished in the absence of extracellular Ca^2+^ (Fig. 4E–H). While 69% of the cells (n = 13) responded in 3 mM Ca^2+^_o_, 0% (n =  11) of the cells responded in 0 mM Ca^2+^_o_ (Fig. 4E). No signal was detected in any of the cells (see average curve in black in Fig. 4G and synchronized average response in red in Fig. 4H).

### The Ca^2+^ oscillations induced by picomolar and nanomolar concentrations have different pharmacological sensitivities

Since oscillating responses represented more than 70% of the responses observed after application of 20 pM EGF (Fig. 2C), we investigated the mechanisms responsible for this type of Ca^2+^ pattern. It was already known that in a variety of cells, activation of EGFR induces a sustained increase in calcium-activated potassium (K_Ca_) channel activity that results in a prolonged membrane potential hyperpolarization [22], [23]. Also, simultaneous EGFR-dependent oscillations of K^+^ channel activity and of intracellular Ca^2+^ have been found [23]. Furthermore, a model of Ca^2+^ oscillation [24] has been proposed, based solely on the dynamic interaction between Ca^2+^ entry and Ca^2+^ activation of K_Ca3.1_ channels. Based on these observations, we tested whether Ca^2+^-activated K^+^ channels could be involved in the oscillatory Ca^2+^ signal observed in response to 20 pM and 2 nM EGF. The application of charybdotoxin (chx), a high affinity blocker of Ca^2+^-activated K^+^ channels, revealed a clear difference (Figs. 4 I–J) in the responses to 2 nM and 20 pM EGF. While no significant change was observed between the proportion of cells reacting to 2 nM EGF (Fig. 4I) in the absence (84%) or in the presence (89%) of 100 nM charybdotoxin, only 27% of cells responded to 20 pM EGF (Fig. 4J) in the presence of the K^+^ channel blocker *vs* 73% in the absence of chx (Fisher exact's test p = 0.006).

## Discussion

### High affinity EGFR activation elicits specific Ca^2+^ signaling

Using sensitive Ca^2+^ imaging, we characterized Ca^2+^signals elicited through high affinity EGFRs. These signals were specific for EGFR activation as ***i)*** when buffer was applied instead of EGF, negligible Ca^2+^_i_ variations were seen (Fig 1C) and ***ii)*** the increase in average Ca^2+^_i_ induced by EGF was inhibited by EGFR-specific neutralizing monoclonal M225 IgGs (Fig. 2E) [12].

### Ca^2+^ oscillations

Using single-cell analysis of Ca^2+^ signals, we were able to demonstrate the oscillating nature of the EGF-dependent Ca^2+^_i_ transients (Fig. 2 A–C) in the majority of the cells responding to 20 pM EGF application. These responses did not occur as a monotonic increase, but as repeated peaks, returning to a basal value, a feature already reported by Cheyette and Gross [25] using fura-2-imaging in A431 carcinoma cells.

Compared to constant Ca^2+^ elevation, calcium oscillations have been shown to increase the efficiency of cell responses [26] by reducing the Ca^2+^ threshold for activating effectors, therefore increasing signal detection at low levels of stimulation. Furthermore, temporal encoding in Ca^2+^ oscillating signal may have a significant impact on the specificity of the cellular response [26] as many Ca^2+^-binding proteins have the ability to transduce different frequencies of Ca^2+^ transients into graded levels of activation (reviewed in [27]). For example it was shown that Ca^2+^ oscillations are optimal signals for Ca^2+^-mediated activation of Ras signaling through the ERK cascade [28]. Moreover, this boosting occurs for Ca^2+^ interspike intervals of 60 s [28], surprisingly similar to the 72 s described in the present report (Fig. 3G). As picomolar EGF concentrations mainly elicit oscillatory Ca^2+^ responses, our data suggest that low EGF concentrations could preserve signal fidelity and specificity with minimum metabolic cost and receptor desensitization, while optimizing information transfer to other signaling pathways.

### Ca^2+^ sources involved in the EGFR response

We showed that high affinity EGFR activation elicits Ca^2+^_i_ variations that are entirely independent of calcium release from internal stores (Fig. 4), as no signal was detectable in the absence of external Ca^2+^. In contrast, at higher EGF concentrations, Ca^2+^ signaling persisted, as previously reported [9], [11]. This would imply that high affinity receptors activate plasma membrane Ca^2+^ channels that are distinct from the store-operated calcium channels, a feature already observed by Zhang and colleagues [21] in a human salivary cell line, while activation of low affinity receptors triggers in addition Ca^2+^ release from internal stores. Our results suggest that the endocrine/paracrine actions of EGF would mainly involve Ca^2+^ flux across the plasma membrane, a mechanism reminiscent of the Mg^2+^ transport triggered by EGF in renal epithelial cells, possibly through TRPM Ca^2+^/Mg^2+^ channels [29].

### Involvement of Ca^2+^-activated potassium channels in the Ca^2+^ oscillatory response

By exploring high affinity EGFR function in Ca^2+^ signaling, we were able to demonstrate, for the first time, the involvement of charybdotoxin-sensitive K^+^ channels. Charybdotoxin is known to block calcium-activated K_Ca1.1_, MaxiK or BK channels [13], K_Ca3.1_ or intermediate channels [14] and voltage-dependent K_v1.3_ shaker current [15]. EGF mediates an increase in K_Ca1.1_ channel activity in vascular smooth muscle cells (VMSC) [30] and controls K_Ca3.1_ channel activation in VMSC [31] and glioma cells [32]. Changes in submicromolar concentrations of internal Ca^2+^ activate calmodulin and gate K_Ca3.1_ channels, which are also regulated by class II phosphoinositide-3 kinase (PI3K, reviewed in [33]). K_Ca3.1_ channels play important roles in the proliferation of lymphocyte T cells [34], vascular smooth muscle cells [31], cardiac pacemaker stem cell development (reviewed in [35]) and tumor cell progression (reviewed in [36]). The K_Ca3.1_-mediated charybdotoxin-sensitive K^+^ current would enhance the electrical driving force for Ca^2+^ entry as suggested for T-cell receptor stimulation [37]. These channels, which are activated at low Ca^2+^ concentrations and undergo desensitization at higher Ca^2+^ level [38], could cause cyclic transient membrane hyperpolarizations and trigger Ca^2+^_i_ oscillations.

### All or none signaling

By performing single-cell Ca^2+^_i_ measurements in response to 20 pM EGF, we were able to uncover a strong heterogeneity in cell responses already reported in A431 cells [25] in a different range of EGF concentrations: although nearly all of the cells were activated at 2 nM EGF, the percentage of responding cells fell to 50% at 20 pM EGF (Fig. 3A); however the cells responding to 20 pM showed Ca^2+^ signals in the same intensity range as those obtained with 2 nM EGF. This heterogeneity in responsiveness suggests pre-existing cell sensitivity, which may arise from the presence of a majority of high affinity receptors in roughly 50% of the cells. EGFRs are allosteric receptors with ligand binding properties that display negative cooperativity, suggesting that the high affinity sites could be a dimeric receptor already preformed and primed for fast activation by EGF. One explanation of our results would be that, in the highly EGF-sensitive fraction of cells, the monomer-dimer equilibrium is shifted towards the dimeric receptor. Consistent with this hypothesis and the observation that high-affinity EGFRs decrease at high cell density [39], fewer cells responded to 20 pM EGF when cells were cultured at high cell density or not on fibronectin-coated coverslips (data not shown), conditions that both influence the monomer/dimer equilibrium.

Quantitative analysis of the oscillating Ca^2+^ responses showed that Ca^2+^ signals at 20 pM EGF are in the same range as those elicited by 2 nM. Median duration (Fig. 3E), area under the first peak (Fig. 3F) and the activation response time (Fig. 3D) changed by only a factor of two to three in response to a two order-of-magnitude variation in EGF concentration. No dose-dependent effect on the interspike interval of Ca^2+^ oscillations (Fig. 3G) was observed. Therefore, it seems that despite variable EGF concentrations and possible variability in receptor expression from cell to cell, a highly sensitive subpopulation of cells is able to produce a robust, almost all-or-none, Ca^2+^ signal in response to EGF application.

### Physiological relevance

Mitogenesis in response to EGF cannot be studied in COS-7 cells as they are partially transformed, but it is known that picomolar EGF doses are able to activate the Ras/extracellular signal-regulated kinase (ERK) signaling cascade, the central driver of cell proliferation in a PI3K-dependent mode, in this cell type [40]. Furthermore, picomolar concentrations of EGF activate selectively ERK and PI3K/Akt pathways while PLCγ, which produces IP3 and triggers Ca^2+^ store release, is activated only by nanomolar EGF concentrations [41]. *In vivo*, low levels of Ras activation stimulate cellular proliferation, while high activation levels induce proliferative arrest in epithelial cells [42]. ERK can be activated by EGF concentrations as low as 2 pM and 40 pM, resulting in proliferation of 8% and 55% of the cells respectively [43]. Moreover, EGFR ligands act on cell proliferation at picomolar concentrations while they display inhibitory effects at higher doses in numerous cells such as carcinoma [44], [45], fibroblastic cell lines [46] and primary keratinocytes [47]. Interestingly, as already commented, oscillatory Ca^2+^ signals with kinetics similar to the ones described in the current study in response to 20 pM EGF seem particularly efficient in triggering Ras/ERK signaling [28]. Altogether, these results suggest that, in addition to inducing a strong Ca^2+^ response, EGF binding to the high-affinity class of EGFRs is able to activate Ras and ERK signaling cascades, and that these pathways may underlie the proliferative effect of picomolar EGF concentrations observed in various cell types. In fact, our results suggest that oscillatory Ca^2+^ signaling induced by physiological EGF concentrations may play a significant role in this process.
